# Evaluating the Impact of Anthropogenic Factors on the Dissemination of Contemporary Cosmopolitan, Arctic, and Arctic-like Rabies Viruses

**DOI:** 10.3390/v14010066

**Published:** 2021-12-30

**Authors:** Andrei A. Deviatkin, Yulia A. Vakulenko, Mariia A. Dashian, Alexander N. Lukashev

**Affiliations:** 1 Laboratory of Molecular Biology and Biochemistry, Institute of Molecular Medicine, Sechenov First Moscow State Medical University, 119435 Moscow, Russia; 2 The National Medical Research Center for Endocrinology, 117036 Moscow, Russia; 3 Martsinovsky Institute of Medical Parasitology, Tropical and Vector Borne Diseases, Sechenov First Moscow State Medical University, 119435 Moscow, Russia; vjulia94@gmail.com (Y.A.V.); alexander_lukashev@hotmail.com (A.N.L.); 4 Department of Virology, Faculty of Biology, Lomonosov Moscow State University, 119234 Moscow, Russia; 5 Faculty of Biomedicine, Pirogov Medical University, 117997 Moscow, Russia; ria.dashyan@gmail.com

**Keywords:** rabies virus, Bayesian, transfer, distances, cosmopolitan RABV, steppe RABV, Arctic RABV, Arctic-like RABV

## Abstract

Rabies is a globally prevalent viral zoonosis that causes 59,000 deaths per year and has important economic consequences. Most virus spread is associated with the migration of its primary hosts. Anthropogenic dissemination, mainly via the transportation of rabid dogs, shaped virus ecology a few hundred years ago and is responsible for several current outbreaks. A systematic analysis of aberrant long-distance events in the steppe and Arctic-like groups of rabies virus was performed using statistical (Bayesian) phylogeography and plots of genetic vs. geographic distances. The two approaches produced similar results but had some significant differences and complemented each other. No phylogeographic analysis could be performed for the Arctic group because polar foxes transfer the virus across the whole circumpolar region at high velocity, and there was no correlation between genetic and geographic distances in this virus group. In the Arctic-like group and the steppe subgroup of the cosmopolitan group, a significant number of known sequences (15–20%) was associated with rapid long-distance transfers, which mainly occurred within Eurasia. Some of these events have been described previously, while others have not been documented. Most of the recent long-distance transfers apparently did not result in establishing the introduced virus, but a few had important implications for the phylogeographic history of rabies. Thus, human-mediated long-distance transmission of the rabies virus remains a significant threat that needs to be addressed.

## 1. Introduction

Rabies virus (RABV) is a negative-sense ssRNA virus belonging to the genus *Lyssavirus*. A number of lyssaviruses can cause a lethal disease called rabies [1]. However, RABV is the only lyssavirus adapted to long-term circulation, not only in bats but also in other mammals, mainly carnivores. Such adaptation is the reason why RABV causes the vast majority of human cases of rabies, with around 59,000 deaths annually [2].

RABV consists of bat-associated and carnivore-associated phylogenetic groups. Bat-associated RABV circulates only among the New World bats. Carnivore-associated RABV may be found in every continent except Australia and the Antarctic [3] and consists of several phylogenetic groups: Cosmopolitan, Africa-3, Arctic-related, Africa-2, Asian, and Indian [3,4,5].

It has been hypothesized that intercontinental transportation of rabid animals by humans provided the global distribution of cosmopolitan RABV, likely over the last few hundreds of years [6,7]. However, it is unclear how systematic human-assisted transfer occurred at different times. Only a few cases of recent long-distance anthropogenic transfers have been reported recently. For example, a virus isolated from a bear in the Russian Far East was genetically most close to viruses collected in European Russia [8]; a sequence of human RABV found in the USA grouped with Brazilian viruses on the phylogenetic tree [9].

Expansion of natural RABV reservoirs is relatively slow. The mean annual virus spread rate depends on the species of the reservoir animal and the ecological region [10]. The rate of rabies spread by rabid foxes in Central Europe was estimated to be 30–60 km per year [11]. The speed of epizootic expansion in Western Siberia steppes was significantly higher, 160–513 km per year [12]. The highest virus dissemination rates were observed in the Arctic region and provided by the long-distance migration of Arctic foxes over the frozen ocean in winter [13,14,15]. This unique ecology of the reservoir host provides a highly dynamic RABV reservoir with virus spread rates of thousands of kilometers per year and almost instant intercontinental transmission of the virus. For example, a rabies virus found in Franz Josef Land (Russia) had a sequence nearly identical to viruses isolated in Alaska (USA) [16]. The origin of this circumpolar Arctic RABV [17,18] reservoir is probably associated with the dissemination of Arctic-like RABV to the Arctic region [19,20]. Currently, Arctic-like RABV is distributed over vast territories in Eurasia, including China, India, Pakistan, Afghanistan, Iran, and Iraq.

Understanding natural virus dissemination patterns is critical for planning preventive measures. Vaccination of humans and domestic animals is an effective measure against the disease. Furthermore, wildlife vaccination can be used to eradicate virus reservoirs in a large territory. Indeed, some parts of the world, e.g., western Europe, have been declared rabies-free areas [21]. Simultaneously, combating against rabies has been less successful in Asia and Africa [2,22]. A single long-distance virus transfer (for example, human mediated) can stall long-term eradication efforts or lead to significant healthcare consequences (reviewed in [23]). However, a systematic exploration of such aberrant transmission events has not been performed.

Historically, finding an RNA virus with a sequence nearly identical to those of viruses from a distant location unequivocally indicates that a long-distance transfer has occurred. In the case of the rabies virus, the simplest explanation is human involvement in pathogen dissemination. This approach may be called the gold standard in the epidemiological investigation of isolated imported cases. However, it is more challenging to analyze the role of anthropogenic dissemination in pathogen evolution and spreading history as a whole, especially as the number of known sequences grows exponentially and goes beyond the capacity of classical analysis tools.

There are several possible approaches to assess the virus spread rate. The first one is based on the dependence between evolutionary distances and the geographical spacing of virus sequences. In that case, all studied objects are analyzed in all possible pairs. Furthermore, evolutionary distances (the proportion of nucleotide sites at which the two compared sequences are different) and geographic distances are calculated for every pair [24]. The results are shown as a scatterplot (referred to as gene-geo plot below). Unlike phylogenetic trees, gene-geo plots do not establish the relationship between individual groups of viruses but show the fingerprint of the geographic and evolutionary distances correspondence. The distribution of dots in this plot may indicate isolated virus transfers or general phylogeographical patterns in the evolution of a virus group as a whole [25].

Another approach, the Bayesian phylogeographic framework, infers virus dissemination velocity within different phylogenetic tree branches. A significant deviation of the inferred spread rates at tree branches would suggest the role of an anthropogenic factor or another possible explanation. This method has two variations: discrete phylogeography that uses isolation locations expressed as fixed categories and continuous phylogeography that uses actual sampling coordinates [26]. A variation of this approach has been applied to characterize RABV spread in North Africa [27]. Continuous phylogeography is highly applicable to distinct RABV subgroups that have been circulating in one main host with a relatively constant ecological pattern [28] but may be less reliable when there are discrete location groups (such as continents) and various hosts with specific ecological traits.

In the current study, we have implemented both the genetic distance-based approach and continuous phylogeography to several RABV groups, i.e., cosmopolitan, Arctic and Arctic-like, to study the role of anthropogenic virus spread on a global scale.

## 2. Materials and Methods

Prior to analysis, sequences represented in GenBank as of June 2020 and annotated as belonging to the species *Rabies lyssavirus* (Taxonomy ID: 11292) were extracted (*n* = 22,628). To provide the best balance of sequence length and number of sequences in the data set, 7616 entries aligning with genome positions 170–1150 in the reference sequence NC_001542 were selected (see below) (Figure S1). The data set was generated using in-house scripts. Then, sequences were aligned using MAFFT v7.310 [29].

Data sets for RABV genome coverage visualization were generated using open-source tools (Supplementary File S1). Then, artificial sequences and sequences with ambiguous positions in the selected 981 nt region were omitted, leaving 7565 genome fragments.

**Figure 1:**
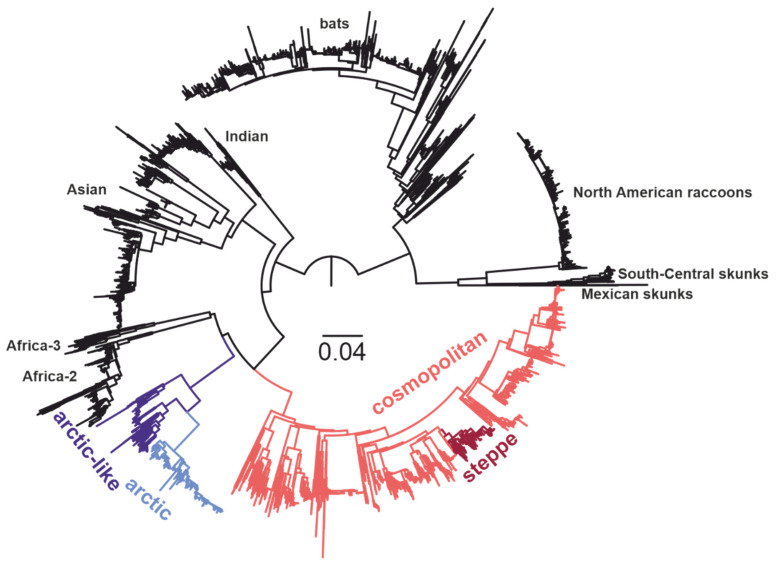
Maximum likelihood tree for RABV (alignment length = 981 nucleotides, number of sequences = 7565). RABV groups analyzed here are indicated by color: cosmopolitan group—pale red; steppe subgroup of the cosmopolitan group—dark red; Arctic-like group—dark blue; Arctic group—pale blue.

Maximum likelihood (ML) phylogenetic inference (Figure 1) was performed using IQ-TREE [30] under the GTR+G4 model (alignment file available at https://raw.githubusercontent.com/AndreiDeviatkin/gene_geo/main/data/fasta/txid11292_260620_950%2B_210-1140_cut_al_final.fas, assessed on 28 December 2021).

Then, cosmopolitan (*n* = 2423) and Arctic-related (Arctic and Arctic-like groups, *n* = 720) RABV sequences were extracted from the whole data set. Furthermore, vaccine-associated sequences were omitted. Arctic and Arctic-like groups were analyzed separately due to suggestively different ecological traits. The steppe subgroup of cosmopolitan viruses was also selected for a separate analysis as one of the best-explored subgroups with a known anthropogenic transfer event.

Finally, only sequences with known dates and locations of sample collection were selected, resulting in the following data sets: cosmopolitan (*n* = 1824), steppe subgroup of cosmopolitan (*n* = 205), Arctic (*n* = 453) and Arctic-like (*n* = 201). Identical sequences with an identical place of sample collection were omitted in the Arctic group before Bayesian analysis, so only 292 out of 453 sequences were used.

Bayesian continuous phylogeographic analysis was performed for the Arctic-like and steppe data sets using the Markov chain Monte Carlo (MCMC) approach implemented in BEAST 1.10.4 [31]. The BEAGLE library v3.1.0 was used to accelerate computation [32]. Prior to analysis, the data sets were tested with TempEst v. 1.5.3 [33] for the presence of a temporal signal.

Maximum clade credibility (MCC) trees were inferred under the relaxed random walk model with a Cauchy distributed diffusion rate variation among branches, which suggests heterogeneity in branch velocities. We used a GTR substitution model with the uncorrelated relaxed log-normal molecular clock assumption [34] and Bayesian skyline population size. MCMC chains were run for 700 and 713 million generations, sampling every 50,000 steps for the steppe and Arctic-like data sets, respectively. MCMC performance was inspected for convergence using Tracer v.1.7 [35]. Maximum clade credibility (MCC) trees were annotated with TreeAnnotator v.1.10.8 using a burn-in of 250 and 71 million generations for the steppe and Arctic-like data sets, respectively. Trees were visualized with FigTree v.1.4.4 [36]. Velocities for MCC tree branches were calculated and then visualized using in-house python script (Supplementary File S2). The same procedure was implemented for control data sets without obvious signs of long-distance transfers.

**Figure 2:**
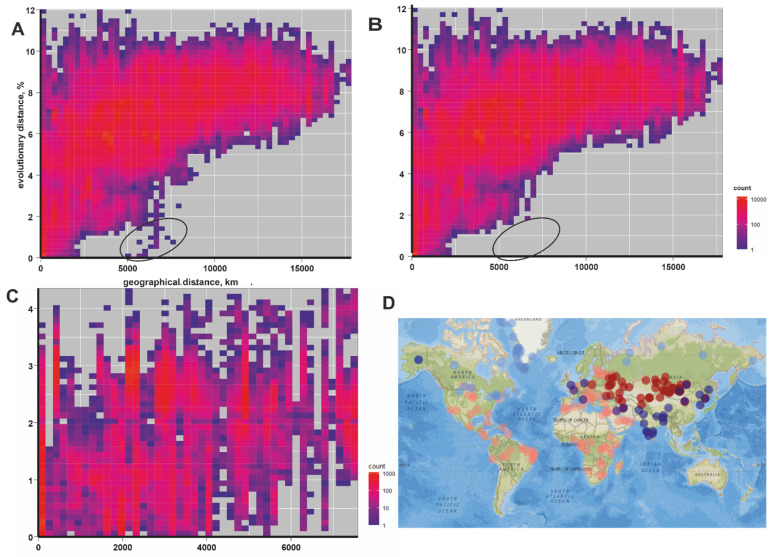
(**A**) Gene-geo plot for cosmopolitan RABV group (*n* = 1824). (**B**) Gene-geo plot for cosmopolitan RABV group without two sequences of viruses transferred between distant regions (#KP997032, #KC737850). (**C**) Gene-geo plot for the Arctic RABV group (*n* = 453). (**D**) The distribution of the cosmopolitan group (pale red), the steppe subgroup of cosmopolitan group (dark red), the Arctic-like group (dark blue), and the Arctic group (pale blue) in the global map. The interactive map with labeled markers is available at https://rpubs.com/andreideviatkin/RABVdistribution (assessed on 28 December 2021).

A map with the locations of sample collection (Figure 2D) was generated in the R environment. The precise coordinates of virus collection sites were not specified in the annotations of most sequences. Therefore, the coordinates of capitals of countries smaller than 1,000,000 sq km were set as places of virus collection. Countries and territories with an area of more than 1,000,000 sq km, i.e., Russia, China, Mongolia, Kazakhstan, Canada, Greenland, USA, Brazil, India, were subdivided into smaller regions according to their administrative regions to infer virus sampling coordinates.

An interactive map with labeled markers is available at https://rpubs.com/andreideviatkin/RABVdistribution (assessed on 28 December 2021). In order to avoid the overlap of isolates from the same region, a slight random noise was introduced into the coordinates used in this map.

Genetic and geographic distance concordances (Gene-geo plots) were visualized in the R environment. Gene-geo plots have not demonstrated consistent statistical patterns for tested data sets. The source code is available at https://github.com/AndreiDeviatkin/gene_geo (assessed on 28 December 2021). This code was implemented in the R shiny webserver as an online tool available at https://andreideviatkin.shinyapps.io/gene-geo/ (assessed on 28 December 2021).

## 3. Results

The genome coverage plot indicated that RABV genome fragments were not evenly represented in GenBank (Figure S1). A 981 nt genome region encoding nucleoprotein N (genome positions 170–1150 in the reference sequence NC_001542) was chosen to provide maximum coverage at a maximum length. Four groups were selected for analysis: two relatively small related groups with different main hosts and suggestively different transfer patterns (Arctic, *n* = 292 and Arctic-like, *n* = 201), the globally prevalent and well-explored cosmopolitan group (*n* = 1824), and its steppe subgroup (*n* = 205), which are well-represented in GenBank and have well-characterized virus trafficking rates [12] and a known long-distance transmission event [16].

Calculation of a Bayesian phylogeographical framework grows with the number of sequences and is not practical for such large data sets. Furthermore, the accuracy of Bayesian phylogeographic models for large data sets remains unclear, as these models tend to perform best at intermediate sequence data set sizes [37]. Thus, the whole cosmopolitan RABV group was analyzed only for the correspondence of geographic and genetic distances (“gene-geo” plot). In addition, the Bayesian phylogeographic framework failed to produce converged parameters for the Arctic RABV data set even after billions of state generations. Thus, this group was also analyzed only by the gene-geo plot.

A gene-geo plot of cosmopolitan RABV demonstrated a fair correspondence between genetic and geographic distances. This relationship was not linear, likely due to mutation saturation at about 10% nucleotide sequence distance and the impossibility of gradual virus spread between continents. The plot contained several sequence pairs that obviously deviated from the general distribution of pairwise values (Figure 2A, circled). These dots correspond to just two putative long-distance transfer events. One was the isolation of a Central Russian steppe lineage RABV from a bear in the Russian region of Primorye (Far East), likely following introduction by a hunting dog [8]. The second one was the isolation of a Brazilian RABV from a patient that immigrated from Brazil to the United States [9]. No other obvious long-distance trafficking events could be suggested from the whole cosmopolitan RABV data set (Figure 2B, the same as Figure 2A, but the two aforementioned “transferred” sequences were omitted).

The gene-geo plot for Arctic RABV demonstrated a limited concordance between genetic and geographic distances distribution patterns (Figure 2C). This lack of a “geographic signal” explains the failure of Bayesian phylogeographic inference in this RABV group. There was evidence of multiple long-distance transfers, i.e., many nearly identical sequence pairs with collection sites separated by thousands of kilometers.

**Figure 3:**
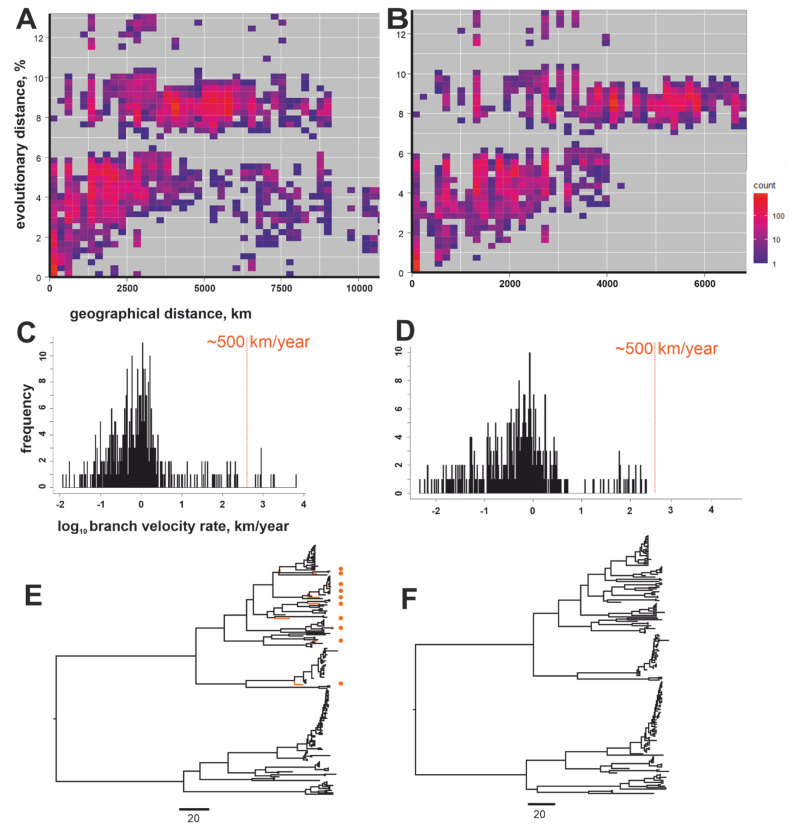
(**A**) Gene-geo plot for the Arctic-like RABV group (*n* = 201). (**B**) Gene-geo plot for Arctic-like RABV group without sequences of viruses apparently transferred between distant regions (#EU836832, #MG011654, #AY956319, #KF154996, #KU963488, #FJ228546, #KY982922, #EF611869, #KY860613). These nine sequences and 16 more sequences that were identical in more than 99.7% of nucleotides and whose collection sites were at a distance of at least 500 km were omitted from the alignment, yielding 176 sequences. (**C**,**D**) The distribution of the inferred velocity rates at the branches of MCC (maximum clade credibility) phylogenetic trees (**E**,**F**) in log-scale for all Arctic-like RABV group sequences ((**C**,**E**) *n* = 201) and Arctic-like RABV group sequences without a putative long-distance transfer history ((**D**,**F**) *n* = 176). The orange line indicates a velocity of 500 km/year. Tree branches with velocity rates over 500 km/year are shown in orange and marked with orange circles.

**Figure 4:**
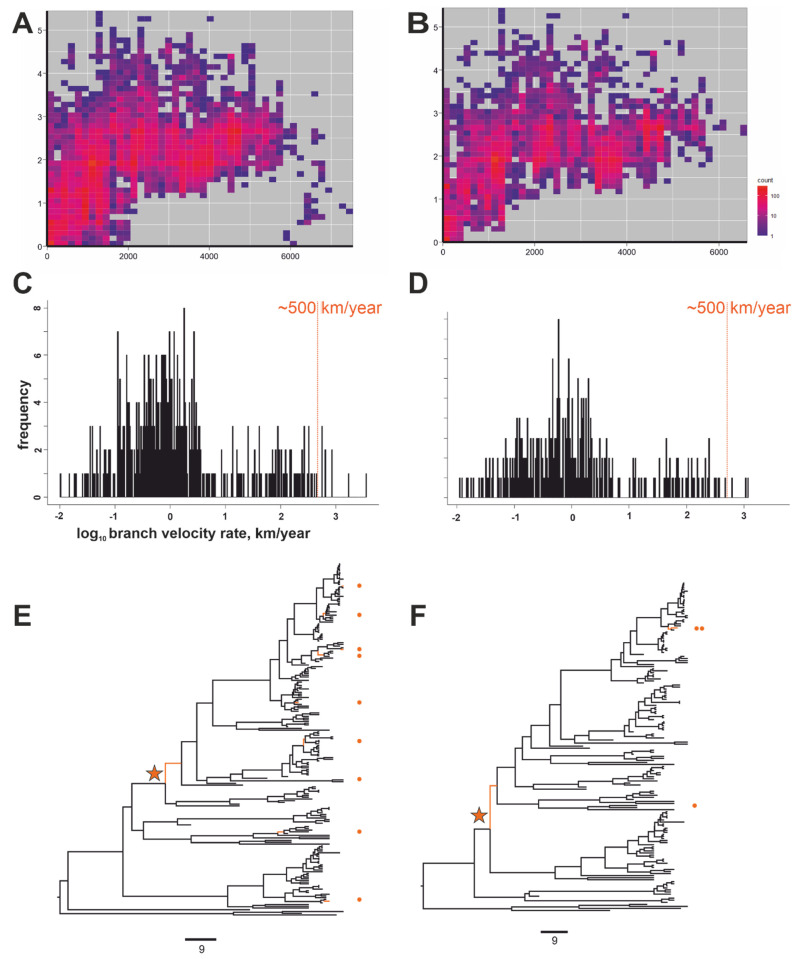
(**A**) Gene-geo plot for the steppe RABV group (*n* = 205). (**B**) Gene-geo plot for the steppe RABV group without sequences that were identical in more than 99.7% of nucleotides and whose collection sites were separated by at least 500 km were removed from the corresponding alignments (*n* = 161). (**C**,**D**) The distribution of velocity rates in the branches of MCC (maximum clade credibility) phylogenetic trees (**E**,**F**) in log-scale for all steppe RABV group sequences ((**C**) *n* = 205) and excluding sequences with a putative long-distance transfer history ((**D**) *n* = 161). The orange line indicates a velocity rate of 500 km/year. (**E**,**F**) MCC phylogenetic trees for all steppe RABV group sequences ((**E**) *n* = 205) and after omitting sequences with a putative long-distance transfer history ((**F**) *n* = 161). Branches with velocity rates over 500 km/year are colored orange and marked with orange circles. The star indicates a transfer from the South Urals region to the east, as discussed in the text.

The distance-based method (gene-geo plot) was tested in parallel with the Bayesian phylogeographic approach for the other data sets, i.e., the steppe subgroup (a group of cosmopolitan RABV, which also included the aforementioned long-distance virus transmission across Russia) (Figure 3) and the Arctic-like group, which also features several suggestive long-distance trafficking events (Figure 4).

The mean nucleotide substitution rate inferred using the N-gene data set for steppe subgroup (*n* = 205) was 4.13 × 10^−4^ (95% HPD 3.24 × 10^−4^–5.00 × 10^−4^) nucleotide substitutions/site/year (s/s/y). This is concordant with the estimates of RABV N-gene substitution rates made for other subgroups of carnivore RABV: 3.89 × 10^−4^ s/s/y (2.88 × 10^−4^–4.96 × 10^−4^) [38], 2.44 × 10^−4^ (2.10 × 10^−4^–2.80 × 10^−4^) s/s/y [3], 3.82 × 10^−4^ (2.62 × 10^−4^–5.02 × 10^−4^) s/s/y [39], 2.47 × 10^−4^ (1.82 × 10^−4^–3.12 × 10^−4^) s/s/y [16], 5.23 × 10^−4^ (3.94 × 10^−4^–6.68 × 10^−4^) s/s/y [40]. This confirmed validity of the Bayesian framework provided a rough estimate for the whole cosmopolitan RABV group substitution rate, suggesting that 1% of substitutions accumulate over 20–50 years.

The gene-geo plots for both the Arctic-like and steppe groups were compatible with recent long-distance virus transfers evident as dots with low genetic and high geographic distances (Figure 3A and Figure 4A). The phylogeographic analysis also found tree branches with velocities over 500 km/year in both RABV groups (Figure 3C,E and Figure 4C,E). To further highlight the capacity of the two methods, known and suggestive “transferred” sequences were omitted from the data sets by excluding sequences identical in over 99.7% nucleotide positions and collected at distances of over 500 km. Additionally, Arctic-like RABV sequences obviously transferred between distant regions were also excluded (e.g., #MG011654 collected in France shared 98% identical nucleotides with #AB699219 collected in Bangladesh). The results of both approaches, i.e., gene-geo plots (Figure 3B and Figure 4B) and the velocity rate distribution (Figure 3D,F and Figure 4D,F), were in concordance. For Arctic-like RABV, this resulted in the absence of “recent transfers” on gene-geo plots and branches with velocity rates greater than 500 km/year upon phylogeographic analysis (Figure 3D,F). However, for steppe RABV, three high-speed (over 500 km/year) branches remained in the phylogenetic analysis of the data sets with manually removed discordant gene-geo sequences (Figure 4D,F). Two branches corresponded to viruses isolated in two large regions, i.e., Russian Zabaykalsky Krai (432,000 sq km) and Chinese Inner Mongolia (1,183,000 sq km). These sequences (#KX533959 and #KY243236) shared 99.6% identical nucleotides (977 out of 981) and thus were not omitted by the artificial threshold. These high-speed branches could be artifacts due to data set limitations because precise isolation locations were unknown and inferred from the coordinates of administrative capitals of these bordering regions. One more high-speed branch that persisted in the purified steppe RABV data set was a deep branch that led to a group of viruses collected in the steppe zone to the east from the Ural Mountains (Russian Southern Siberia, Russian Far East, Mongolia, Northern China) (Figure 4F, indicated by an asterisk). This finding suggests a human-assisted transfer of steppe RABV to the east from the Ural Mountains that occurred between 1958 and 1961 (95% highest posterior density (HPD): 1944–1978). This suggestive human-assisted transfer was not evident in the gene-geo plot (Figure 4B).

The most vivid examples of sequences classified as “cosmopolitan” or “arctic-like” that had evidence of long-distance transfers upon gene-geo analysis are summarized in Table S1. As long-distance transfers of the Arctic group viruses are apparently systematic, data on individual sequences was not shown in Table S1. Roughly half of these long-distance events have been reported previously. In two cases, the branch velocity was not notably affected, likely because it could be spread among several branches by the statistical algorithm. It is noteworthy that a stringent distance cut-off was used; thus, less prominent long-distance transfers could have been overlooked. In addition, in this work, a high-throughput approach was used without an in-depth analysis of individual cases and without consideration of geographical and political barriers (state borders, water bodies, transportation networks, etc.); therefore, these findings should be treated with care. In two more cases, long-distance transfers were detected by the gene-geo plots but could not be verified by Bayesian phylogeography because the non-reduced cosmopolitan data set was too large.

## 4. Discussion

In general, RABV is considered a typical object for phylogeographic studies [41]. However, in the Arctic region, virus transmission speed was strikingly different from this classical pattern and was so high that it precluded a statistical phylogeography analysis. The case of Arctic RABV indicates that a phylogeographic signal should be checked to ensure correlation between geographic and genetic distances, and thus the applicability of a data set for a phylogeographic analysis. Gene-geo plot does so similarly to classical verification of a temporal signal prior to doing a statistical phylogenetics analysis involving a molecular clock. This is conventionally performed by assaying the correlation of root-to-tip distances in a sequence-based phylogenetic tree with isolation dates, for example, using TempEst (formerly Path-O-Gene) software [33]. One key difference in the verification of a temporal and geographic signal in a sequence data set is that an object cannot go back in time, but host migration trajectories are complex, and a virus can be carried back to its place of origin. Thus, the formalization of “geographic signal” in genomic data requires further studies.

While the Bayesian frameworks converged for other RABV groups, potential human-mediated transfers were a ubiquitous factor to be considered according to the gene-geo plots. We used a stringent cut-off of 500 km/year and a comparable genetic distance criterion to define likely anthropogenic transfers. This approach detected several known events and a comparable number of undescribed ones (Table S1). Overall, about 10–20% of all sequences bore signs of long-distance transfers. The actual fraction of affected viruses could be even higher because a 500 km/year cut-off was chosen arbitrarily, and there are even more sequences with branch rates above 300 km/year, which are also poorly compatible with a natural spread of steppe RABV. Of course, this number does not represent the actual proportion of human-assisted virus transfers because sequences from cases of medical and veterinary significance are overrepresented in GenBank. However, it is clear that in both Arctic-like and steppe RABV, unnatural long-distance transfers remain a significant factor in rabies epidemiology. In addition, some RABV strains demonstrated resistance to neutralizing mAbs and vaccine-induced antibodies [42].

It has been discussed previously that the history of RABV was significantly shaped by human activity. The worldwide dissemination of the carnivore-associated group of RABV was associated with colonial expansion and international trade accompanied by uncontrolled dog transportation that occurred in the 15th–19th centuries [3]. The role of long-distance transfers in recent RABV evolution is less clear. The predominantly gradual virus spread confined by natural barriers was described in both a global data set [4] and in country-wide studies, for example, in China [43]. The gene-geo plots suggest just three intercontinental transfers in both the cosmopolitan and Arctic-like RABV groups. Two events, one in the cosmopolitan group (Brazil-USA) and one in the Arctic-like group (India-Alaska), had been described previously [9,44]. The third case, to the best of our knowledge, has not been discussed yet. Isolate with GenBank #KU963488, collected in Alaska, USA, and #KX434489, collected from buffalo’s brain in Andhra Pradesh, India, shared 98.8% identical nucleotides (969 out of 981). Other related sequences were found in Asia; thus, the direction of this transfer was obviously from Asia to Alaska. Isolate #KU963488 was found in a wild animal (Arctic fox). This means that the anthropogenic transfer of RABV from India to Alaska led to spill-over to a novel host. At the same time, there were no other sequences of that Arctic-like subgroup in Alaska. Considering that #KU963488 was collected in 2007, it is likely that the Arctic-like group of Indian origin was not fixed in Arctic foxes after this particular introduction. It is noteworthy, though, that the Arctic group suggestively originated from the Arctic-like group upon virus introduction to circumpolar regions hundreds of years ago [19].

In the cosmopolitan group, all virus pairs spaced by over 8000 km also differed by at least 5% in the nucleotide sequence. Extrapolating RABV substitution rates identified here and elsewhere (see above), this corresponds to 100–250 years of circulation after the most recent common ancestor. Thus, virus control measures in the last century were generally efficient to prevent virus spread between the Old and New World, and intercontinental RABV transfer is not a major factor in virus evolution nowadays.

The majority of recent long-distance virus trafficking found in this study occurred within Eurasia (Table S1). Moreover, there could be more such events because our definition of long-distance transfers in terms of gene-geo distances and inferred branch velocities was arbitrary and could even underestimate their impact. One such event, suggested by statistical phylogeographic analysis and dating about 60 years ago, could have led to virus spread in East Siberia. This finding is concordant with long-distance transfers involving the steppe and Arctic-like groups that were noted in China but presumably occurred over 100 years ago, according to the presented phylogenetic trees [43]. One historical example that shows a possible mechanism of such human-assisted virus transfer was the translocation of thousands of raccoons from Florida, USA, to Virginia, USA in 1977–1981, which could have resulted in the emergence of raccoon rabies in the middle Atlantic states of the USA [23,45]. There was no evidence that recent (less than 30 years ago) virus transfers resulted in the establishment of the introduced lineages of cosmopolitan and Arctic-like RabV in Eurasia. In addition, few to none such recent long-distance (over 1000 km) transfers could be suggested from the phylogenetic analysis provided for Iran and China [42,46]. However, human-mediated RABV spread could have shaped the epizooty in Brazil at the end of the 20th century [47], is common nowadays in Tanzania [48], and was the major factor driving the current epizooty in Indonesia [49]. In North Africa, recent long-distance virus transfers have been noted but were hardly the main route of virus spread [27]. Thus, intracontinental long-distance transfers remained an important factor of RABV evolution in the mid-20th century in many locations. Currently, virus trafficking control is efficient in some countries but lacking in others. Moreover, the current data set is relevant only for countries with sufficient resources to isolate and sequence the virus. In contrast, less affluent countries may remain entirely out of the scope of such studies.

The two methods for the detection of aberrant long-distance virus transfers produced comparable results but had significant differences. Statistical phylogeography found many known and unreported long-distance transfer events but failed in the cases with an absent phylogeographic signal (Arctic RABV) or a too high number of sequences (cosmopolitan group). Gene-geo plots were applicable in both these cases to detect either a few long-distance transfers in the cosmopolitan group (Figure 2A) or to characterize the Arctic data set as a whole (Figure 2C). Data set reduction [50] can be used to circumvent the data set size limitation, but this should be performed with care because it may introduce unexpected biases in statistical phylogenetic calculations. However, the gene-geo plot failed to detect non-recent events, as exemplified by the putative cross-Urals transfer detected in the steppe group only by statistical phylogenetics (Figure 4E,F). Gene-geo plots do not provide statistical significance of the findings. However, the statistical support from Bayesian frameworks may be misleading if improper priors were used. In the absence of an independent reference method, there is no way to assess the validity of the predictions. It can be thus suggested that using methods complementing each other may produce more precise results. Long-distance movements are rare or even non-existent in some RABV groups but common in others. Anthropogenic transfers have been investigated epidemiologically on many aforementioned occasions. Herein we evaluated different methods for analyzing them and providing an overview of their effect on the evolution of distinct RABV groups.

A major limitation of the large-scale phylogeographic analysis is the lack of precise virus sampling coordinates in GenBank records. Dedicated studies can use exact sampling location data; however, most published sequences have just country or, at best, province data. Many regions are hundreds of kilometers across, and virus isolation coordinates had to be approximated. Transfers between such regions could appear more rapid than they actually were. On a global scale, we observed a fair correlation between genetic and geographic distances. However, a more precise analysis, such as inferring virus trafficking rates, may not be reliable with the available data. In addition, for this reason, we abstained from analyzing less obvious potential long-distance transfers. Clearly, precise sampling coordinates would significantly increase the value of sequence data.

Another general limitation to ecological studies using RABV genomic data comes from the over-representation of cases of medical or veterinary importance in sequence databases. These events generally represent endemic viruses in the area but are, in most cases, dead-end events in virus evolution. Indeed, most long-distance virus transfers described here did not result in the establishment of novel virus lineages in distant regions. Only one suggestive transfer that occurred less than 70 years ago actually significantly impacted steppe RABV phylogeography (indicated by a star in Figure 4E,F).

## 5. Conclusions

The human-mediated spread of rabies is a significant threat (reviewed in [23]) and requires preventive countermeasures. Molecular surveillance for rabies allows for detecting long-distance virus transfers even in areas that are already endemic. A simple and easily automatable gene-geo analysis demonstrated suitable correspondence with sophisticated phylogeographic methods and may be a valuable tool for routine surveillance and a supplementary method for dedicated phylogeographic studies.

## Data Availability

The data and source code presented in this study are openly available in the GitHub repository https://github.com/AndreiDeviatkin/gene_geo (assessed on 28 December 2021). This code was implemented in the R shiny webserver as an online tool available at andreideviatkin.shinyapps.io/gene-geo/ (assessed on 28 December 2021).

## Supplementary Materials

The following supporting information can be downloaded at: www.mdpi.com/article/10.3390/v14010066/s1, Figure S1: RABV genome layout and RABV genome fragments longer than 950 nucleotides represented in GenBank. Purple lines indicate the genome region used for this study.; Table S1: Selected closely related viruses found at distant locations; File S1: in-house script for RABV genome coverage visualization, File S2: in-house python script for MCC tree branches velocities calculation and visualization.
