# Supplementary material for: Evaluating the Impact of Anthropogenic Factors on the Dissemination of Contemporary Cosmopolitan, Arctic, and Arctic-like Rabies Viruses
Source: Viruses. 2021 Dec 30;14(1):66. doi: 10.3390/v14010066 (PMC8777955; doi:10.3390/v14010066)
Supplement: Supplementary file 1 [file viruses-14-00066-s001.zip › Figure S1.pdf]

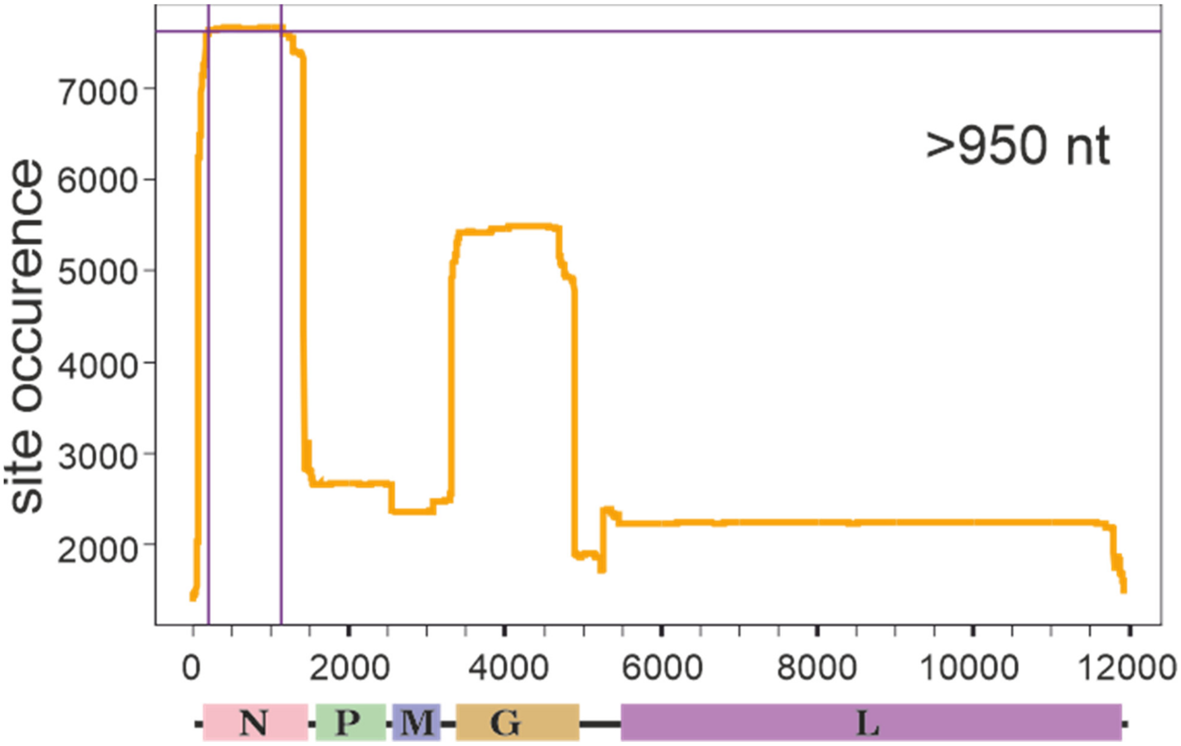

Figure S1. RABV genome layout and RABV genome fragments longer than 950 nucleotides represented in GenBank. Purple lines indicate the genome region used for this study.
