# Supplementary material for: Evaluating the Impact of Anthropogenic Factors on the Dissemination of Contemporary Cosmopolitan, Arctic, and Arctic-like Rabies Viruses
Source: Viruses. 2021 Dec 30;14(1):66. doi: 10.3390/v14010066 (PMC8777955; doi:10.3390/v14010066)
Supplement: Supplementary file 1 [file viruses-14-00066-s001.zip › File_S1.pdf]

```
makeblastdb -in ~/work/rabv_geo/NC_001542.fas -dbtype nucl -title NC_001542 -out
~/soft/blast+/db/NC_001542; cd ~/soft/blast+/db ; blastn  -max_hsps 5 -max_target_seqs 1 -word_size
7 -query ~/work/rabv_geo/txid11292_260620_annotated_950+.fas -db NC_001542 -evalue
0.0000000001 -out ~/work/rabv_geo/blastn_txid11292_260620_annotated_vs_NC_001542_tmp -
outfmt  '6 qseqid sseqid pident length qlen slen evalue qstart qend sstart send'  ; cd ~/work/rabv_geo ;
cut -f 1,10,11 blastn_txid11292_260620_annotated_vs_NC_001542_tmp > rabv_coord_in_NC_001542  ;
cut -f 2,3  rabv_coord_in_NC_001542 > tmp; sed -i 's/\t/-/g' tmp ;  perl -pe 's/(\d+)-
(\d+)/join(",",$1..$2)/eg' tmp > tmp1 ; sed -i ':a;N;$!ba;s/\n/,/g' tmp1 ;  sed -i 's/,/\n/g' tmp1 ; sort -g
tmp1 > tmp2; sed -i '/^$/d' tmp2;  uniq -c tmp2 > tmp3 ;  sed -i 's/^ *//g' tmp3;  awk '{print $2,$1}' tmp3
> GB_occurence_per_NC_001542_sites_950+.txt ;
```
