# Supplementary material for: Evaluating the Impact of Anthropogenic Factors on the Dissemination of Contemporary Cosmopolitan, Arctic, and Arctic-like Rabies Viruses
Source: Viruses. 2021 Dec 30;14(1):66. doi: 10.3390/v14010066 (PMC8777955; doi:10.3390/v14010066)
Supplement: Supplementary file 1 [file viruses-14-00066-s001.zip › Table S1.pdf]

Table S1. Selected closely related viruses found at distant locations.

Genbank reference number and isolation location	Nearest Neighbor in Terms of Evolutionary Distance that was collected in a distant region	Approximate Direct Air Distance, km	Nt Sequence Identity between Strain of Interest and Its Nearest Neighbor, %	Branch velocity rate, km/year	Information about long-distance transfer
		**Arctic-like**			
FJ228546, USA, Alaska, 2007*	KF660246, India, Gujarat, 2012	9740	98	734	[39]
KY982922, China, Qinghai, 2014	KC171645, South Korea, 1999	2778	100.0	207	this study
KX148226, Afghanistan, 2002*	EF611869, Iraq, 2005	2029	99.9	889	this study
KY860613, Russia, Primorsky Krai, 1989**	EF611868, Russia, Zabaikalsky krai, 1980	1693	100.0	156	this study
KU963488, USA, Alaska, 2007*	KX434489, India, Andhra Pradesh, 2002	10146	98.8	897	this study
MG011654, France, 2016*	MG099711, Bangladesh, 2016	7856	100.0	6377	Specified in GenBank annotation
EU836832, Italy, 1996*	KX148228, Nepal, 1998	6522	99.7	865	[40]
AY956319, Germany, 2005*	KY775603, India, Gujarat, 2014	5933	99.7	1882	[41]
KF154996, United Kingdom, 1987*	LT909541, Pakistan, 1984	6180	99.0	654	[42]
MH258833, India, Goa, 2014*	MH258817, India, Chennai, 2014	713	100.0	1536	this study
		**Cosmopolitan**			
MF197741, Poland, 2008	KX533960, China, Inner Mongolia, 2015	6609	98.2	N/A (several branches between these sequences)	this study
MK598340, Hungary, 2007	LC422733, Mongolia, Selenge province, 2018	6018	97.1	N/A (several branches between these sequences)	this study
KR781563, Brazil, Rondonia, 2007	KU938904, Peru, 1999	1486	99.8	N/A (not Steppe subgroup)	this study
KY002894, Russia, Novosibirsk, 2002*	KT965738, Kazakhstan, Almaty region, 2014	1383	99.8	839	this study
KJ958263, Russia, Tuva, 2012 *, ***	KX533961, China, Inner Mongolia, 2015	1790	99.8	656	this study
KP997032, Russia, Primorsky Krai, 2014*	MF574191, Russia, N.Novgorod, 2013	6048	100.0	3621	[7]

KC737850, USA, Massachusetts, 2011	KR781569, Brazil, Acre, 2007	5763	100.0	N/A (Not Steppe subgroup)	[8]
LC422737, Mongolia, Selenge, 2018*	LC422736, Mongolia, Bulgan, 2018	1163	100.0	550	this study
LC422738, Mongolia, Khuvsgul, 2018*	LC422742, Mongolia, Tuv, 2018	529	100.0	639	this study
AB571018, Mongolia, Ulaanbaatar, 2005*	AB571015, Mongolia, Govi Altai, 2005	823	100.0	855	this study
KJ958241, Russia, Krasnoyarsk, 2011*	KJ958230, Russia, Khakassia, 2011	271	99.69	560	this study
KY243236, Russia, Zabaikalsky Krai, 2015*	KX533959, China, Inner Mongolia, 2014	1253	99.59	1721	this study

* The velocity rate of the branch in the phylogeographic tree between this pair of samples was less than 500 km/year.

** These 100% identical sequences were isolated in 1989 and 1980, respectively [43,44], and should be treated with caution regarding possible artefacts.

*** These viruses were isolated in neighboring regions, thus the evidence of long-distance transfer could be an artefact of approximate sampling coordinates.
